# Synergy and timing: a concurrent mass medical campaign predicted to augment indoor residual spraying for malaria

**DOI:** 10.1186/s12936-019-2788-9

**Published:** 2019-05-06

**Authors:** Richard C. Elliott, David L. Smith, Dorothy C. Echodu

**Affiliations:** 1 Micron School of Materials Science and Engineering, Boise State University, Engineering Building, Suite 338, Boise, ID 83725 USA; 2 Pilgrim Africa, 115 N 85th St #202, Seattle, WA 98103 USA; 3 Institute for Health Metrics and Evaluation, University of Washington, 2301 Fifth Ave., Suite 600, Seattle, WA 98121 USA

**Keywords:** Malaria, Vector control, IRS, MDA, MSAT, Synergy, Spraying, Ross–Macdonald models, *Openmalaria* simulation

## Abstract

**Background:**

Control programmes for high burden countries are tasked with charting effective multi-year strategies for malaria control within significant resource constraints. Synergies between different control tools, in which more than additive benefit accrues from interventions used together, are of interest because they may be used to obtain savings or to maximize health impact per expenditure. One commonly used intervention in sub-Saharan Africa is indoor residual spraying (IRS), typically deployed through a mass campaign. While possible synergies between IRS and long-lasting insecticide-treated nets (LLINs) have been investigated in multiple transmission settings, coordinated synergy between IRS and other mass medical distribution campaigns have not attracted much attention. Recently, a strong timing-dependent synergy between an IRS campaign and a mass drug administration (MDA) was theoretically quantified. These synergistic benefits likely differ across settings depending on transmission intensity and its overall seasonal pattern.

**Methods:**

High coverage interventions are modelled in different transmission environments using two methods: a Ross–Macdonald model variant and *openmalaria* simulations. The impact of each intervention strategy was measured through its ability to prevent host infections over time, and the effects were compared to the baseline case of deploying interventions in isolation.

**Results:**

By modelling IRS and MDA together and varying their deployment times, a strong synergy was found when the administered interventions overlapped. The added benefit of co-timed interventions was robust to differences in the models. In the Ross–Macdonald model, the impact compared was roughly double the sequential interventions in most transmission settings. *Openmalaria* simulations of this medical control augmentation of an IRS campaign show an even stronger response with the same timing relationship.

**Conclusions:**

The strong synergies found for these control tools between the complementary interventions demonstrate a general feature of effective concurrent campaign-style vector and medical interventions. A mass treatment campaign is normally short-lived, especially in higher transmission settings. When co-timed, the rapid clearing of the host parasite reservoir via chemotherapy is protected from resurgence by the longer duration of the vector control. An effective synchronous treatment campaign has the potential to greatly augment the impact of indoor residual spraying. Mass screening and treatment (MSAT) with highly sensitive rapid diagnostic tests may demonstrate a comparable trend while mass LLIN campaigns may similarly coordinate with MDA/MSAT.

## Background

With 76% of the world’s entire malaria morbidity burden borne by just 13 nations, and malaria prevalence related to both micro and macrocroeconomic indicators of poverty, those countries most burdened tend also to be those least able to afford its suppression [1–4]. As GDP growth and malaria are negatively correlated [2], national malaria control programmes and their partners in endemic countries must manage effective malaria control within stringent budget constraints. And this problem is getting more severe: spending per person at risk in the highest burden countries has reduced even further in the last three years, creating challenges for continued global progress towards malaria eradication [1]. Though effective interventions to stem transmission are relatively expensive, the cost of failing to control malaria is higher, both in lost health and in hindered economic growth [3, 4]. Prudence and good strategy are necessary to balance up-front with ongoing costs for malaria control.

Elimination, however lengthy the journey may be, is the most important goal for all control programmes. As the only stable result that avoids the burden of continued, intensive control efforts, it also limits or avoids acquired resistance in parasites and mosquitoes. The human and economic costs of maintaining elimination are also lower than those associated with achieving it, so it is desirable to reach elimination quickly. Malaria control programme designers in high burden countries face the challenge of finding the fastest, most effective, and least expensive route from endemic disease to pre-elimination and from pre-elimination to elimination, while applying continual downward pressure on transmission. Provided that no resurgence occurs, and that costs can be contained within budget limits, it is axiomatic that optimal routes to elimination are those that lower transmission as rapidly as possible [5]. Countries with high or moderately high transmission have special interest in cost-effective control combinations capable of producing large, rapid reductions in transmission.

Synergy in malaria control is the interaction of two or more control interventions that produces a combined impact greater than the sum of their separate effects. Synergies have the potential to increase health impact per dollar spent in a malaria control strategy, and therefore to save programmes money. Possible synergies in integrated vector management, principally between indoor residual spraying (IRS) and long-lasting insecticide-treated nets (LLINs), have been eagerly explored for this reason. Unfortunately no strong, super-additive advantage to using both of these vector interventions together has yet emerged [6–9], and even where the combination appears somewhat more effective than either intervention alone the effect is only found at certain transmission intensities [8]. Potential synergies with novel control methods have also been examined [10]. Somewhat surprisingly, synergies between vector control campaigns and medical campaigns have not been well explored from a theoretical and quantitative point of view, although there has been some consideration of potential synergies and/or antagonisms between LLINs and vaccines [11].

Any control interventions exhibiting synergies with the most common forms of vector control could create large cost savings by maximizing the impact of the interventions. This is particularly true for IRS due to its high programmatic cost in insecticide and labour. In 2015, 106 million people were protected by IRS, 49 million of them in Africa. Since IRS is effective in medium to high transmission areas, [12–14]), while at the same time expensive, the decision to use it often depends on available funding [15, 16]. IRS coverage in Africa actually dropped to 45 million people in 2016, despite the increasing use of more effective next generation insecticides, due to programme concerns around cost [1]. Synergies between IRS and other control tools could maximize the health impact per cost of this effective intervention by deepening its impact.

Besides the widespread use of LLINs and IRS for vector control, endemic countries also invest heavily in anti-malarial medicines, primarily artemisinin-based combinations (ACT) for the treatment of disease. These medicines also act as powerful control tools to reduce transmission [17, 18]. In 2016, more than 196 million ACT doses were distributed by sub-Saharan NCMPs in the public sector [1] and the majority of these ACT doses were not distributed via mass campaigns, but rather intermittently and continuously through case management of uncomplicated malaria [19].^1^ Given the large combined global investment in medical and vector control, it is worth exploring possible cooperation between these two sets of tools. Synergies between IRS and the mass synchronized use of effective malaria medicines are particularly attractive, because high burden communities already see high use of such medicines. From a resource perspective, MDA in such a setting might be considered a “rearrangement” of effective treatment courses otherwise consumed in case management. In this way, a relatively small investment of labour might augment the impact of an existing IRS programme, preventing infections otherwise requiring intensive case management and treatment.

IRS is almost always applied with a mass campaign, in which coverage is expanded population-wide within a short period of time, and initiates effective, yet impermanent control; these campaigns are dynamic in time. Consequently, synergies between the deployment of vector control campaign, IRS, and a mass medical campaign, whose dynamics of control are also time-dependent, are explored. Of note, the distribution of LLINs is also almost always applied via mass campaign, and the same reflections around the dynamic nature of protection apply to this ubiquitous form of vector control as well. Recently, a robust synergy between these campaigns, MDA with a concurrently deployed IRS campaign, was noted and theoretically quantified [20]. As both the IRS and MDA campaigns exert control over transmission differently, and with different durations, this synergy is dependent on their relative times of deployment. Below, these interventions are deployed at differing separations in time and at different transmission intensities, in order to illustrate and quantify any super-additive impacts of deploying general vector control together with a mass medical campaign of anti-malarials. Though it is not explicitly modelled below, it should be noted that mass screening and treatment (MSAT) is a campaign-style medical intervention of the same type as MDA. MSAT impact on the reservoir of parasitaemia depends on the sensitivity of the diagnostics used, but will generally be lower than MDA. MSAT, however, may well be more palatable to national control programmes or to communities than MDA, and if highly sensitive RDTs are used to detect parasitaemia, the impact of a high coverage MSAT will approach that of an MDA campaign. Similarly, an IRS campaign is modelled below, though the results are likely applicable for comparable campaign style form of vector control, in particular LLINs with short-lasting insecticides and/or a fast attrition rate.

It is recognized that MDA is more successful in the presence of vector control [21]. Though potential synergies between MDA and IRS, and their dependence on timing, have not previously been rigorously quantified, the combined impacts of a joint campaign have nevertheless historically been recognized and employed. A notable example is the Garki project, which investigated many aspects of transmission and the effects of some control strategies on various transmission intensities [22, 23]. IRS and MDA were deployed both as isolated campaigns and together. When IRS and MDA were deployed in tandem, at high coverage (85%), the campaigns achieved a high level of control, “the prevalence of parasitaemia decreased very rapidly and varied in the 1–5% range, according to season [22].” Prevalence was in many cases suppressed to very low levels, although elimination was not achieved. Similarly, combined MDA and LLIN campaigns in Henan Province in China in the late 1990s and early 2000s were shown to be generally effective in maintaining a stable low rate of infection [24]. Sustained interruption of transmission has been reported from combined use of IRS and MDA in highland Uganda in 1960 [25], and intriguingly also from combined MDA and LLIN campaigns in Vanuatu [26]. Of course, many more isolated MDA [27–29], and IRS campaigns [30, 31] have been carried out more recently, with varying successes, and recently reviewed for the Asia Pacific region [32] and Africa [33, 34]. The most common vector control efforts in Africa, both LLINs and IRS, generally focus on endophagic vectors, with outdoor/residual transmission largely overlooked. The role of outdoor transmission is expected to become increasingly important as vector behaviour changes and prevalence drops, but for many high burden areas, it seems that the endophagic vectors play a critical role for transmission, and will be the focus below.

Recent investigations in sub-Saharan Africa indicate large short-term prevalence reductions from a comprehensive MDA campaign, especially in low transmission environments [29, 35]. These lend some justification to the current WHO recommendation for MDA, namely, that it be used either in areas approaching elimination or for control of epidemics in a time-limited sense (such as during complex emergencies like the recent Ebola crisis) [36]. However, the WHO guidelines call for more implementation research and explicitly support the use of modelling to guide the optimum use of MDA in a programme setting. In particular, the increased health impacts and/or reductions in costs that could be obtained by co-deploying vector control together with an MDA or an alternate mass medical campaign like MSAT warrant careful examination.

All high transmission settings require vector control. Both IRS and LLINs are recommended for use in high transmission settings [37]. IRS can be even more effective than LLINs at these intensities [12, 13], particularly when potent, long-lasting insecticides are used [14]. Often, IRS strategy involves transmission reductions achieved through years of repetitive sprayings [38], and as IRS is a resource intensive intervention, such multi-year efforts are expensive to achieve. Below, the use of an MDA campaign as an amplifier or “accelerant” for a jointly deployed IRS is explored; if the addition of MDA greatly augments the impact of an IRS campaign and allows reductions to be achieved with fewer rounds of spraying and/or fewer treatment courses of ACT, significant cost savings may ensue. A comparable accelerating or amplifying role for a medical campaign was suggested by Macdonald [39], “There is no reason to suspect that the adequate use of potent insecticides, if properly checked, should not result in the elimination of African malaria in its most stable form, but the cost in insecticide and labour is bound to be high[er] ...Economy is to be sought by the combination of insecticidal attack with such methods as effective mass treatment to reduce the period of operation.”

The proposed use of a carefully timed MDA (or MSAT) as a potential vector control accelerant in a high transmission setting also differs conceptually from its use in an elimination setting. Communities saddled with high transmission experience high ACT use as a matter of course, and in some areas consume more than one ACT dose annually per capita. Given the chemical pressure already extant in highly infected communities from frequent case management, an MDA or MSAT in such settings may not necessarily increase overall ACT use, but instead could be seen as a temporal and demographic reorganization of existing ACT consumption. Rather than planning to employ MDA or MSAT campaigns for a long period of time, starting from high transmission and continuing until elimination is achieved, a co-deployment of MDA or MSAT together with LLINs or IRS could be used for short periods (several rounds of each, perhaps) to achieve much-needed deep reductions to a “new normal.” This new low might afterwards be sustained by integrated community case management and ongoing vector control.

Modelling helps to generate an understanding of the mechanisms of transmission control, and to uncover potential synergies [20, 40, 41]. Recent modelling efforts have investigated similar control interventions and combinations [19, 42–45]. Generally, campaign-style control measures are carried out as finite programmes that are deployed, have a prescribed duration, and then promptly end. Post-campaign (with exception), the modelled community rebounds to its previous transmission balance where malarial infections are again prevalent. As such, below, elimination scenarios are not considered directly but the process of large-scale reductions in the host and vector reservoirs are examined as an important component of the elimination pathway.

## Methods I: Interventions in a variant of the classical model

Medical and vector-based campaigns address different stages of the transmission cycle. Medical campaigns such as an MDA affects and diminishes the human reservoir of parasitaemia, and contrastingly, vector control campaigns target mosquitoes and deplete the vector reservoir. During an MDA campaign, a community’s host reservoir is cleansed; the MDA kills parasites, and offers all those covered a limited prophylactic period of perhaps 2–4 weeks depending on the anti-malarial used. Transmission is directly affected in proportion to coverage as fewer hosts harbour parasites that are passed on to the mosquito, and the momentum of host infection$\rightarrow$vector transmission$\rightarrow$new host infection is slowed. Biting however continues undeterred.

An IRS campaign provides a different mechanism of control: there is no therapeutic aspect and transmission is instead decelerated through the reduced biting from a diminished vector population. These interventions are complementary to each other in that they target the two sequestered parasite populations, those of the host and the vector. As both these reservoirs refill at certain rates, it is natural to wonder what combination and timing of reservoir cleansing of parasites in humans and mosquitoes is capable of producing the largest reduction in malaria.

To investigate these questions, and to compare different methods of suppressing infections in a model community, both a Ross/Macdonald model variant and *openmalaria* simulations are employed. And specifically, the combination of MDA with IRS in very generic transmission contexts is considered in both modelling efforts. Given their importance in moderate to high transmission settings, only relatively potent interventions are modelled, and this analysis is limited to a consideration of effective, high-coverage campaigns. The isolated and combined impacts of these interventions are investigated for a few different transmission settings.

Interventions in the Ross/Macdonald model are simply incorporated as control efforts temporarily reducing transmission via the reproductive number, $R_0$. The MDA and IRS campaigns are considered first independently, which serves to introduce their different mechanisms of bottlenecking transmission. Writing $R_0=bcC/r$, the basic reproductive number is the rate infections invade the community *bcC* divided by the rate *r* they depart. *C* is the vectorial capacity and *b* and *c* are transmission efficiencies mosquito-to-human and vice-versa; hosts recover from infection at an average rate of *r*. Thus, during a campaign that modifies one or both of these rates, the reproductive number changes, $R_0\rightarrow R_0^{I}$, with the superscript *I* referencing the reproductive number during an intervention. Reductions may be achieved through enhanced mosquito mortality during an IRS which affects *C*, or through (say) diminished human infectious periods from a medical campaign, which changes *r*. Interventions modeled below are just finite periods of bottlenecked transmission, and this is a tremendous simplification and a useful approximation. The periods of intervention activity/duration are estimated, and dynamical trajectories for the host and vector infectious populations are calculated through the intervention periods with $R_0^I<R_0$ and associated parameters, described in more detail below. When the intervention concludes, the transmission intensity immediately reverts back to pre-intervention levels, restoring $R_0^I\rightarrow R_0$, also an approximation. The system subsequently relaxes according to these ambient conditions. At this time, transactions of parasites between human hosts and mosquitoes return without the dynamical constraints set by the intervention, albeit with depleted parasite reservoirs. As is well known [20, 46], the Ross/Macdonald dynamical system may relax to only one of two stable points: the trivial equilibrium of elimination, or to a stable transmissive environment where parasites are exchanged freely and there are measurable populations of infectious hosts and mosquitoes. In all cases considered below, post-intervention the system relaxes to this latter fixed point, and elimination is not considered.

### Mass drug administration

At its simplest, transmission is affected two-fold during an MDA campaign, reduced first through the accelerated rate infections depart a community with an administered anti-malarial, and second, through chemoprophylaxis. Both of these effects directly modify the reproductive number $R_0^I$ during the effective period of the intervention and change the dynamical course of the infectious populations. The first of these is an alteration of the human infectious period $r^{-1}\approx 150$ days [47], whose average wanes with the campaign. During an MDA with good coverage much of the host population has their carried parasite load extinguished and thus overall has shortened infections, reducing $r^{-1}$. This is accomplished in the Ross/Macdonald variant with a rate amplification, $r\rightarrow \xi r$, with $\xi >1$ for the effective period of intervention. The second alteration regards chemoprophylaxis, the protective period of maybe 2–4 weeks (set below to be 2 mosquito lifetimes), where mosquito-to-human transmission is greatly reduced due to the protection afforded by the anti-malarial. This is set by a second parameter reduction $b\rightarrow b/\mu$, corresponding to a forced reduction in mosquito-to-human transmission. Consequently, these two alterations greatly reduce the reproductive number, $R_0\rightarrow R_0/\mu \xi$ for the short duration of the intervention, and the campaign’s effect size is $R_0/R_0^I=\mu \xi$ [44, 48, 49]. Just after the effective time period of the MDA, these effects promptly expire and the parameters *r* and *b* return to their pre-intervention values. Appendix A has more details on system dynamics and intervention effects during the campaigns.

**Fig. 1:**
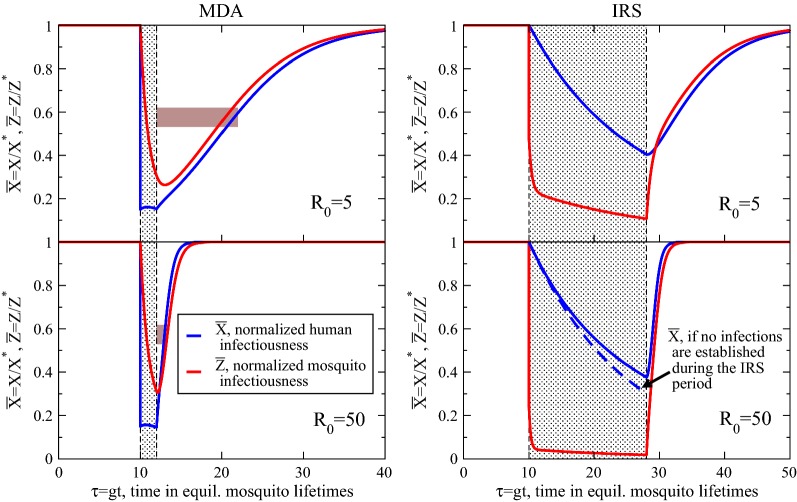
Normalized trajectories for human ${\bar{X}}$, and mosquito ${\bar{Z}}$ infectiousness with an applied, model MDA or IRS at (nondimensional) time $\tau =10$. The application period of the interventions is highlighted in the panels. The upper panels display the longer-lasting effects of campaigns in lower-transmission environments, $R_0=5$, while the lower panels show the much shorter recoveries for moderate transmission environments, $R_0=50$. Bars on the left indicate the half-life estimates of Eq. 1 for regaining pre-intervention equilibria. The dashed line in the lower right shows a limit for the vector control explained in the text

As might be expected of a medical intervention, these modifications for the MDA campaign do not directly alter the governing dynamics of infectious mosquitoes. The campaign solely modifies the host parasite reservoir and has repercussions in the vector only through subsequent, preempted transmission. This can be clearly seen in Fig. 1 (left panels) where the effects of a model MDA campaign in the Ross/Macdonald variant are shown for a low ($R_0=5$) and moderate ($R_0=50$) transmission setting. In the figure, the host infectiousness trajectory *X*, and sporozoite rate *Z*, are normalized by their pre-intervention equilibrium values, ${\bar{X}}=X/X^*$ and ${\bar{Z}}=Z/Z^*$, so they both initiate and, upon retraction of the intervention, bounce back towards equilibrium at ${\bar{X}}, {\bar{Z}}\rightarrow 1$. These trajectories thus indicate percent reductions in parasitaemia in the host or vector. On the abscissa, time *t* is measured in mosquito lifetimes, $\tau =gt$. It is clear that during the prophylactic period of the MDA campaign, a gray-highlighted region, with a diminished reproductive number maintained at $R_0^I=0.5$, system dynamics decay temporarily towards elimination. The initial 85% reduction in ${\bar{X}}=X/X^*$, is prominent, corresponding loosely to achieved reductions with $\sim$ 85% campaign coverage (compliance and adherence to the prescriptive treatment is here assumed, and drug resistance is neglected). Notably, though, the sporozoite rate drops almost as quickly, i.e. the vector parasite reservoir depletes in just a few mosquito lifetimes. This is an important, first dynamical consequence of the effects of a medical intervention: the sporozoite rate is labile with respect to changes in the host reservoir. The MDA campaign targets and mostly clears the large reservoir of parasites in hosts and this quickly echoes in the much smaller vector reservoir; the carried parasite load of the ephemeral mosquito population readily adjusts to changes in the host parasite reservoir.

At $\tau =12$ in Fig. 1, when the prophylactic period expires, host protection is promptly lost. Mosquitoes continue to bite hosts though clearly fewer are infected and those individuals who were previously protected are eligible to become infected once again. A resurgence takes place and the stable equilibrium of the pre-intervention transmission setting, ${\bar{X}},{\bar{Z}}\rightarrow 1$, is re-equilibrated in time. This property may be considered a resilience of the system: the propensity for an endemic setting to return to stable, widespread parasitaemia after a dynamical perturbation, which is here the MDA campaign. Re-equilibration takes much more time in the low-transmission $R_0=5$ setting than the higher transmission one ($R_0=50$), perhaps an obvious result, but if MDA is used in isolation from a vector control intervention it clearly has a more lasting impression in a low transmission or elimination-ready context.

Employing the approximation mentioned above that the transmission environment immediately reverts back to pre-intervention settings post-campaign, i.e. setting $R_0$ promptly back to its prior (pre-intervention) value, enables a linearized stability analysis. A characteristic time to re-establish the pre-intervention equilibrium may be found. The time to regain half the number of infectious individuals of the community’s previous stable equilibrium is approximately [20],

1 $$\begin{aligned} \tau _{m}\approx g f_I^{-1}(X^*) \log \left( 1+{\bar{X}}_f^{-1}\right) , \end{aligned}$$

where $f_I(X)$ is the force of infection, the rate at which successful, infectious bites are received per host. It is a function of the infected host proportion at the intervention’s expiry [46], and of the stable transmission setting prior to the intervention. The logarithm in Eq. 1 boosts the characteristic time by a factor related to the depth of infection suppression achieved by the intervention. The host prevalence at the conclusion of the intervention (at $\tau =12$ for the MDA in Fig. 1) is denoted ${\bar{X}}_f$, and indicates that the lower host prevalence can be driven, i.e. smaller ${\bar{X}}_f$, the longer it takes to re-establish the equilibrium level of parasitaemia. Thus, more effective MDA campaigns (or other intervention) require longer times to establish prevalent infections.

Equation 1 indicates the restoration rate, the resurgent rate malarial infections invade the community, is essentially the force of infection of the pre- and post-intervention entomological setting: the rate of viable infectious bites of the system’s stable entomological state. It sets the (perhaps obvious) pace in which malarial infections invade the populace, after the intervention’s control has been exerted. Equation 1 simplifies with, $f_I(X^{*}) \approx {r R_0}/{(1+\gamma )}$ ($\gamma$ is the average number of bites in a mosquito’s lifetime that infects it) so that $f_I\sim R_0$, and the characteristic time of restoration (Eq. 1) scales as $\tau _m\sim R_0^{-1}$, as has been previously noted in SI models [50, 51]. Thus, high transmission intensities become parasitaemic faster post-intervention than do low ones, a sensible trend seen easily in Fig. 1. Bars in the left MDA panels indicate this approximate time to recover half the infections in the populace. More details of this analysis, which is again based on a linearized approximation, can be found in reference [20].

The characteristic time of resurgence in Eq. 1 as an approximation is most relevant for small changes in the infectious densities, which clearly in Fig. 1 they are not. This estimate is however not without merit given that they illuminate the attributes of transmission that are most responsible for the resurgence. It is worth noting that the most conspicuous difference with $R_0$ in Fig. 1 is the variation in recovery times for the two transmission settings post-intervention, a result emphasized with the indicated half-lives. The scaling of this return to prevalent malaria in Eq. 1 with $\tau _m\sim {R_0}^{-1}$ indicates the relative recoveries differ by a factor of ten, and this matches the figure extremely well. In passing, it is worth mentioning a final interesting property of this relapse time set by $f_I^{-1}$: it does not endlessly shorten with $R_0$ but in fact saturates, ultimately achieving an asymptote $g^{-1} f_I(X^*)\approx 1+\gamma$ (for large $R_0$), an intriguingly $R_0$-independent result. This is a consequence of the fast turnover of the vector population, their success in infecting hosts is ultimately limited by their short lifecycle [20].

It is furthermore important to recognize another limitation of this admittedly crude approach here. In a real setting the transmission does not instantaneously revert back to pre-intervention levels, as has been assumed here, but more likely grows as a given intervention deteriorates. For this matter, the estimates of Eq. 1 should be regarded as a fastest estimate for this characteristic resurgence time. This point will be revisited below for relevant comparisons with simulation. Furthering this point, partial host immunity and heterogeneous host selection are overlooked details in this analysis, and they too should serve to lengthen the resurgence time.

### Indoor residual spraying

An IRS campaign is an interesting counterpoint to MDA, in which the local mosquito population is quickly and dramatically reduced from exposure to applied insecticide. It may be taken as somewhat of a dynamical inverse to a mass medical campaign: while synchronized medical treatment depletes the host reservoir, vector control should serve to reduce the vector parasite reservoir. In both cases, a sudden depletion in one reservoir forces the response of the other. During mass medical treatment, it was seen above that the vector responds very quickly to an immediate alteration in host parasitaemia, and a fast plummeting infectious vector proportion resulted. In just a few mosquito lifetimes, the sporozoite rate falls and becomes more commensurate with the infectious host proportion. Based on this, a natural question arises: how does the host reservoir dynamically respond to the sudden depletion of the vector reservoir?

To register the killing effects of a vector control campaign, the death rate of mosquitoes must get boosted during the campaign. Amending this with a simple population model [20, 52] enables the approximation that a simple boost in mosquito mortality, $g\rightarrow \kappa g$ ($\kappa >1$), establishes an equilibrium of a smaller population of mosquitoes, and does so on a timescale shorter than a mosquito lifetime, $(\kappa g)^{-1}$ [20]. Working within this population model, a diminished but static population of mosquitoes results: the vector continues to be replenished at the same rate but mosquitoes live shorter lives during the active period of the campaign. As a consequence, the reduction in the reproductive number during an IRS campaign is,

2 $$\begin{aligned} R_0 \Rightarrow R_0^I=R_0\frac{[P_e]^{\kappa -1}}{\kappa ^2}, \end{aligned}$$

where $$P_e=e^{-gn}$$ is the survivorship of mosquitoes after the latency time *n*. The scaling of the reproductive number in Eq. 2 with $$\kappa$$ has been discussed previously [46]. Transmission is suppressed as $$\kappa ^2$$ in the denominator with one power from the reduction in the mosquito population, and a second from their shorter expected lifespan. The factor of $$P_e^{\kappa -1}$$ results from the fewer older mosquitoes present during the campaign that enable further transmission. Other than the coverage-related initial reduction and the campaign duration common to all interventions, it should be noted that the IRS evolution is configured with only a single parameter, $$\kappa$$ (or $$R_0^I)$$, which alone changes the dynamical course of the system during the IRS period of bottlenecked transmission.

The dynamical effects of this model IRS campaign for two transmission settings are shown in Fig. 1. There is a marked reduction in ${\bar{Z}}$, synonymous to the initial reduction in ${\bar{X}}$ for the MDA, which is set by ${\bar{Z}}(\tau _0)=(1-c_0)+c_0{P_e}^{\kappa -1}$, with coverage $c_0$ taken to be the percentage of all (relevant, proximal, host-seeking) mosquitoes affected by the intervention initiating at $\tau _0$ [20]. The intervention duration is set comparable to that of a typical insecticide, much longer for the IRS than the immediate cleansing of an MDA campaign, and set here to ${\bar{\tau }}=18$ (or $18g^{-1}\approx 180$ days), a time window highlighted in the figure. There is also no prescribed decline in the efficacy in the insecticide; it is effective during the $18g^{-1}$ period of the IRS, and then subsequently inactive. For a direct comparison with the MDA modeled above, the augmentation of the mosquito mortality rate is again set to preserve $R_0^I=0.5$ for all transmission environments, a requirement that determines $\kappa$. The net killing effect disproportionately affecting older mosquitoes forces a diminished sporozoite rate during the effective period of the IRS, seen in the figure, and the system temporarily attracts to the stable node of elimination, ${\bar{X}},{\bar{Z}}\rightarrow 0$. Prevalent infectious densities then return post-intervention when the community is subject to re-infection from the fast dynamics of the recovering vector parasite reservoir. ${\bar{Z}}$ quickly bounces back towards the proliferation stable point and host infectiousness follows (${\bar{X}}, {\bar{Z}}\rightarrow 1$), the resurgent response rate scaling with the ambient force of infection as before with the characteristic time of Eq. 1.

A striking contrast with the MDA campaign here is that the host infectiousness ${\bar{X}}$ responds slowly to changes in the sporozoite rate ${\bar{Z}}$ during vector control. Fast changes in ${\bar{X}}$ result in fast changes in ${\bar{Z}}$ in the MDA above, but the reverse is not true. Figure 1 depicts the slow decline in host infectiousness after the mass killing of infectious mosquitoes during the IRS. In fact, the sporozoite rate plummets for the effective duration of the insecticide (here roughly half a year), and host infectiousness responds with a slow, consistent decay. This asymmetry is a result of infections lost only through their expiry at the natural host healing rate, *r*, something directly modified by the MDA but summarily untouched by vector control. For comparison, the lower right panel of Fig. 1 also shows the decay in host infections in the limit of a vanishing inoculation rate, or when zero successful host infections take place in the active time of the IRS. It is drawn with a dashed line, and represents the limit of a perfectly effective vector control campaign. In short, the mechanism of protection offered by the IRS is to isolate the host reservoir from replenishment, and it is only its long duration that allows host infections to expire and clear, causing ${\bar{X}}$ to wane. The IRS period, as modeled here, has only a trickle of new infections due to a strongly suppressed mosquito population, but with no mechanism or program in place to clear existing host infections, waiting for them to heal at their slow rate is the only means to a reduced presence of malaria in the community.

In summary, while the fast clearing of host infections in an MDA campaign results in a correspondingly fast clearing of the vector reservoir, the cleansing of the vector reservoir with an IRS does not result in the fast clearing of the host reservoir. Host infections are untouched with the vector control, and forced to clear on their own natural timescale of $r^{-1}$. In other words, the flux of malaria out of the community is still slow despite a heavy modification of the vectorial capacity, which greatly reduces its flux in. This indicates the needed coordination of medical and vector control efforts: the protection of a depleted host reservoir with vector control may enable powerful gains.

### Synchronous IRS and MDA

**Fig. 2:**
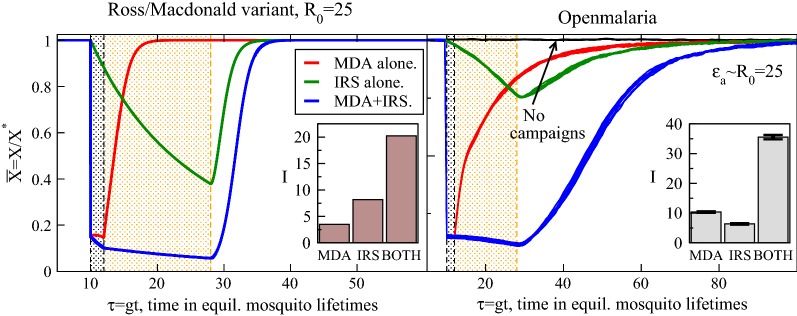
Normalized trajectories for host infectiousness ${\bar{X}}$ with an applied, model MDA, IRS, or both, at (nondimensional) time $\tau =10$. The application period of the interventions is again highlighted in the panels. The left panel is for the Ross/Macdonald theory variant and the right for *openmalaria* with indicated transmission intensities. For MDA + IRS campaigns jointly administered, gains from the MDA quickly clearing the host reservoir are sustained by the offered protection of the IRS. The insets show the intervention impacts *I* as defined in the text, Eq. 3, indicating the superior suppression of infections for synchronous MDA + IRS

Given that the host and vector parasite reservoirs react differently to the interventions, and especially in a rather complementary manner, it is natural to next consider their joint deployment. Host prevalence trajectories for the synchronous deployment of an IRS and MDA are plotted in Fig. 2 for the Ross/Macdonald theory and for *openmalaria*, which will be discussed in more detail below. Focusing first on the Ross/Macdonald variant, synchronous deployment has both a depleted infectious host proportion and reduced sporozoite rate for 85% coverage, in correspondence with the individual campaigns considered above. The interventions are identical to those described in the subsections above, with the same durations and parameter reductions common to those individual interventions. Here, dynamics are first determined from the joint campaigns, for $10\le \tau \le 12$, and just after they evolve with the IRS, as its period of efficacy continues beyond the MDA, to $\tau =28$. Intervention durations are indicated on the figure as before. As with the cases above, at the moment of expiry of both campaigns, the parameters revert back to those of transmission prior to the interventions. Equilibrium is reestablished asymptotically, scaling as dictated by Eq. 1.

Prevalence is driven essentially to the pre-elimination regime for either the Ross/Macdonald theory or *openmalaria* in Fig. 2 with joint campaigns, reminiscent of the control reported by the Garki project [22]. But this figure also shows the coordinated means to obtaining this control. First, host infectiousness is initially reduced by the high coverage MDA, and rather than this control being the short-lived improvement that the MDA campaign achieves alone, these gains are prolonged by the IRS. Vector control provides the needed protection for a cleansed host reservoir; the initial depletion of host infections established by the MDA is effectively sustained by the IRS. Resurgence is delayed until the vector control has faded.

In order to measure this effect, a simple metric of the fitness of an intervention (or sequence of them) is the number of host infections prevented through the course of its effective time. Establishing this as a prevalence reduction, rather than an absolute count of infections, this impact is,

3 $$\begin{aligned} I=\int d{\tau } [{\bar{X}}_0(\tau )-{\bar{X}}_c(\tau )], \end{aligned}$$

where ${\bar{X}}_0(\tau )$ and ${\bar{X}}_c(\tau )$ are the prevalence trajectories for the community without interventions and with them, respectively. ${\bar{X}}_c(\tau )$ with subscript *c* denotes a trajectory with included campaigns. Impact measures for the Ross/Macdonald variant further simplify with ${\bar{X}}_0(\tau )=1$, the prevalence absent interventions is that of the average of the transmission setting. It is written generally here because it will be applied to simulations below where noise is present and ${\bar{X}}_0(\tau )\ne 1$, instead fluctuating about unity. This impact is the area in Fig. 2 bound by the trajectories with campaigns, ${\bar{X}}_c(\tau )$ and those without, ${\bar{X}}_0(\tau )$, and is simply interpreted as the percentage of infections prevented by the campaign times its effective time. As such, a short duration campaign that deeply cleanses the host reservoir, such as a high coverage MDA in a high transmission setting, may have a net impact *I* comparable to a different, low coverage intervention with a correspondingly long duration (perhaps a bednet distribution at modest coverage). The impact *I* measures the total abilities of the intervention(s) to stem malarial infections. By inspection alone, the impact shown in Fig. 2 (and the insets) for joint campaigns exceeds that of the individual interventions, and even their sum, indicating the coordination of their effects mentioned above.

## Methods II: *Openmalaria* simulation of MDA, IRS and LLINs

The right panel of Fig. 2 shows sets of trajectories for *openmalaria* simulations, which are separately run as a comparison for these interventions. Juxtaposed with the semi-analytic Ross/Macdonald model, these trajectories and impacts provide an interesting comparison, in particular a novel setting for testing the synergy, which is in fact immediately apparent in this figure. These simulations model transmission and interventions entirely differently but arrive at remarkably similar conclusions.

Briefly, *openmalaria* is an agent-based simulator that relies on a more sophisticated model of transmission and includes elaborations such as demographic heterogeneity, partial immunity in the populace, the role of case management in health systems, and variable transmission/infectivity based on a list of factors [53–60]. Notably, the evolving, extrapolated load of parasite densities in inoculated hosts is monitored, and from it immune status and host infectivity is inferred. The global entomology of a simulation and various attributes of the vector(s) are also separately configurable. Further discussion of many of these features can be found in these references, and an Appendix B details many of the settings of the simulations run here; the particular focus here is on a comparison with Ross/Macdonald theory. These elaborations of the *openmalaria* model that make it unique are also not turned off, which would serve to compare base models of transmission (and interventions), but are rather deliberately included, within reason, to look in particular for the resilience of this synergy in different modelling environments. For example, case management is present (though it is very minimal), and acquired partial immunity is included, though clearly neither of these is present in the Ross/Macdonald model. The intention here is to see if an independent approach to the modelling yields a comparable signature of intervention impact and timing. Adhering to general cases and some conventions for all demography, entomology and interventions in simulation yields a reasonable comparison. And as indicated in Fig. 2, some features of the interventions are in correspondence with the semi-analytic model.

Again, Fig. 2 (right panel) shows, for these *openmalaria* simulations, the three situations described above: an isolated MDA, an isolated IRS, and their synchronous deployment. Eight trajectories are plotted for each set indicating the dispersal of the prevalence profiles, though as drawn they mostly overlap on the displayed ordinate scale. The transmission intensity is set to an annual entomological inoculation rate (EIR) of $\mathcal{E}_a=25$ bites/host*annum with no seasonal variation, based on a correspondence with the reproductive number from a separate investigation [61]. The prevalence trajectories are normalized in the same manner as the Ross/Macdonald theory, which requires running separate simulations to determine the average prevalence in the community without interventions. These simulations are run to establish $X^*$ (and $Z^*$ if desired), the equilibrium values for the infected host fraction, absent interventions. These normalized simulation trajectories of Fig. 2 are for hosts with any parasite density in their blood (those with 0.01 parasites/$\upmu$L or greater) and not for patent hosts, a convention set for an apt comparison with the Ross–Macdonald model where hosts either harbour parasites or do not. Intervention coverage is set to 85% with their durations as specified above and indicated on the figure. No pattern of insecticide decay is specified for the IRS in the simulation, but it is rather generically configured as a step function, on during its effective period and then abruptly off. More details can be found in Appendix B.

## Results

Infection suppression with combined vector control and MDA campaigns modelled here is notably better than additive, an effect seen easily in Figs. 1 and 2. Their impacts for an isolated MDA, isolated IRS, and synchronous MDA + IRS are shown in insets to the figure and demonstrate the enhanced suppression of the combined, jointly-administered interventions. In the Ross/Macdonald variant, roughly twice as many infections are prevented by a joint deployment of IRS and MDA in comparison with an isolated MDA plus an isolated IRS campaign.

A comparison of *openmalaria* simulations with the Ross–Macdonald variant shows that the recovery times, post-intervention, are much longer for the simulation. The scale of the abscissa for the simulations is twice that of the Ross/Macdonald variant to accommodate the slower resurgence of parasitaemia in the simulated community. The Ross/Macdonald analysis (Eq. 1) indicates the community regains half of its infected proportion for $\tau _m\approx 3$ ($R_0=25$) post-intervention. In contrast, these simulations predict a half-life of resurgence several times longer: at nearly three months post-intervention ($\tau \approx 20$ for the MDA campaign alone) the Ross–Macdonald theory indicates the effects of the MDA are essentially gone while it takes perhaps more than a year for the equivalent equilibration in *openmalaria*. Malaria invades the community post-intervention at a much slower rate in *openmalaria* simulation, as noted above. Since the relapse time of Eq. 1 is a fastest estimate, this is not unexpected. The force of infection recovers and slowly accelerates in the *openmalaria* simulation in this time period, while it is assumed to revert immediately to pre-intervention levels in the Ross/Macdonald analysis. Partial host immunity also slows resurgence, as was explored earlier [20]. While the recovery rate is expected to be slower in simulation, the question of whether it (or any other resurgence rate) is a good quantitative estimate, especially within the context of non-spatial modeling, is uncertain.

The addition of a co-timed MDA to an IRS campaign in Fig. 2 augments its impact by roughly three (in Ross/Macdonald) or four (in *openmalaria*) times, depending which method is used to model the IRS. The difference is due to the fact that the *openmalaria* campaign for IRS is plainly not as effective as the Ross–Macdonald variant. As mentioned above, the rate *r* at which host infections heal primarily determines the effective rate of infection loss in ${\bar{X}}$ during the IRS, as (typically) only a trickle of new infections impact the rate. In the *openmalaria* simulation, infections appear either to expire more slowly, or to initiate more frequently during the IRS period. More details of the IRS parameterization in *openmalaria * are given in Appendix B, though the IRS potency is maximized in this application, with pre- and post-prandial mortality affecting essentially all contacted mosquitoes. The heterogeneity of host selection with a given, input demographic, is an included sophistication of the *openmalaria* transmission model, and this may play a role in weakening the modelled IRS campaign here. Regardless, with either model, the large augmentation of IRS impact upon addition of an MDA is striking.

### Impacts of the interventions at variable transmission intensities

**Fig. 3:**
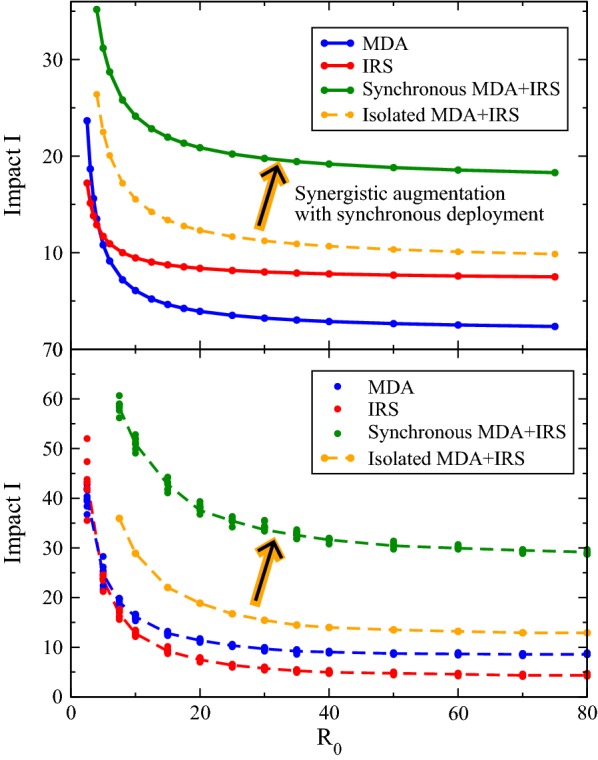
Intervention impacts *I* from the Ross/Macdonald semi-analytic theory (top) and *openmalaria* (bottom) as a function of transmission intensity. In the upper panel, the solid lines show the impacts for the MDA and IRS alone, and together, when synchronously deployed. A dashed line indicates the impact of an IRS and MDA when applied temporally in isolation, consisting of the sum of the two isolated campaigns. Synchronous deployment far exceeds this impact, and may be understood mechanistically in Fig. 2, where the initial cleansing of the host reservoir from an MDA is maintained by the IRS. In the lower panel, *openmalaria* simulations show the same trends, though impacts are mostly greater since the recovery times post-intervention boost their effective duration. For each $R_0$, eight simulations were run and their impacts plotted, though for most intensities the overlap is significant compared with the point-size. The connecting lines are drawn to guide the eye, and are used to interpolate the dashed profile for the isolated MDA + IRS, in accordance with the upper panel. One apparent contrast is the very weak IRS in *openmalaria* compared to the semi-analytic model. All interventions have 85% coverage, as before

Impacts are compared in Fig. 3 for a high coverage MDA and IRS with varying transmission intensity $R_0$. The top panel shows impacts from the Ross/Macdonald model and the lower from *openmalaria*. Again, the insets of Fig. 2 show bar charts of the impacts *I* from the prevalence trajectories in the figure, and these correspond with those in the top panel of Fig. 3 for $R_0=25$. Impacts at other transmission intensities $R_0$ are calculated and assembled for this figure and, for example, indicate the strongly variable effects of the MDA, as apparent in Fig. 1. As recovery times shrink with growing $R_0$ (resurgence is faster in higher transmission settings), the impact $I(R_0)$ of any intervention wanes. And while the recovery time reaches an asymptote—mentioned above, this rebound time $\tau _m$ asymptotes—this causes the impact of each campaign to correspondingly saturate, an effect readily seen in Fig. 3.

An IRS campaign is found to be generally more robust than the MDA (with exception for $R_0\lesssim 5$) in the semi-analytic theory, with static intervention transmission intensities of $R_0^I=0.5$, but is also relatively invariant with transmission setting. Its impact at $R_0=20$ is rather comparable to that at $R_0=75$. The impact decay of an isolated MDA with $R_0$ is far more apparent. It is very assertive at low $R_0$, something that has been recently noted [29, 35].

Also shown in Fig. 3 (top) are two profiles of combined interventions intended for comparison. The first shows the impact of the joint, synchronous deployment of an MDA with an IRS; this is always greater than the impact of the (dashed) profile below it showing the additive impact for these two interventions applied at separated times. This is the synergy of these interventions, illustrated in Fig. 2 for $R_0=25$, but shown here to be present at essentially all transmission intensities. The impact with synchronous deployment always exceeds, and almost doubles, that of two isolated campaigns.

Corresponding *openmalaria* simulations are run with and without interventions to calculate the impact $I(R_0)$ for this transmission model, and are shown in the lower panel of Fig. 3. For each $R_0$, eight simulations are run with and without the indicated intervention, and the impacts of Eq. 3 summed. For each intervention, or their combination, all eight values of $I(R_0)$ are displayed on Fig. 3 though for larger $R_0$ their points mostly overlap on the figure appearing as a single point. The stochastic noise is present throughout, though only plainly visible for low $R_0$; the variance about equilibrium is greater in these settings resulting in a noisier impact $I(R_0)$. Both single intervention MDA/IRS impacts have connecting lines on the figure to guide the eye, which are simply averages over the eight simulations intended only to facilitate an apt comparison with the upper panel. The sum of these averages is shown in the isolated MDA + IRS profile, which is an extrapolated net impact of two isolated campaigns.

The signature of synergy is also apparent in these simulations, and is similar in character to that exhibited in the Ross/Macdonald variant above. As a comparison, the values of the impacts are first generally greater in the simulations, a result of the slower recovery times post-intervention, and this augments the effective duration of any intervention and its impact *I*. Notably, also in the comparison of models, the IRS is weaker for *openmalaria* at essentially all transmission intensities and as a result, an MDA combined with an IRS in *openmalaria* confers an especially striking advantage when compared with IRS alone. On the other hand, the MDA in *openmalaria* is largely similar in appearance to the Ross/Macdonald in the top panel, with its assertive control at low $R_0$ and strong $R_0^{-1}$ decay. Its comparatively longer recovery in the simulation results in an impact that at large $R_0$ asymptotes to a higher value than those in the Ross/Macdonald theory above. The IRS campaign, though weaker than the MDA at nearly all transmission intensities, intriguingly is comparably strong to the MDA for low $R_0$ settings. In either case, the synergy of these interventions is found and is of nearly the same magnitude: a doubling (or more) of the impact of the interventions. A strong synergy manifests at all transmission intensities for both modelling techniques.

### Timing medical and vector control interventions: synchronous or near-synchronous deployment boosts impact

With all transmission parameters and environmental conditions equal, Fig. 3 seems to indicate that the synchronous application of vector and medical control via a campaign style intervention yields roughly twice the combined impact of isolated campaigns, and from three to four times the impact of solitary vector control campaigns. It is clear that infected host populations are robustly suppressed with a synchronous deployment of complementary mass medical and vector control interventions.

To see whether this synchronous deployment is in fact the impact maximum, a deployment time of the IRS campaign is fixed in the middle of a window of duration $\tau _{tot}$ of time, at $\tau =0$, for a few transmission settings, and the deployment time of the MDA is systematically changed. A sweep of deployment times is performed for MDA campaigns carried out far enough in advance of the vector control campaigns to be (essentially) fully in isolation of the effects of them, to far enough post-campaign to again be fully isolated. These limits of separation should naturally agree for isolated campaigns. The synergistic effect found above with their combined application will have some signature, which is sought below through a comparison of their impacts, varied for deployment times.

A fractional impact of these two interventions, deployed synchronously or not, is measured through a comparison with a benchmark case: the same transmission setting with both interventions applied temporally in isolation. For the Ross–Macdonald variant, a trajectory with entirely isolated MDA and IRS campaigns, ${\bar{X}}_{iso}(\tau )$, is first run with interventions separated by several years so that the system has essentially relaxed to the fixed points of Eq. 7 before, between and after the individual interventions. The fractional improvement with a given timing, with respect to the isolated campaigns, is,

4 $$\begin{aligned} \chi _s(\tau _0)={I_c(\tau _0)}/{I_{iso}}=\frac{ \int d\tau [{\bar{X}}_{0}(\tau )-{\bar{X}}_{c}(\tau )]}{\int d\tau [{\bar{X}}_{0}(\tau )-{\bar{X}}_{iso}(\tau )]}, \end{aligned}$$

where again ${\bar{X}}_0(\tau )$ and ${\bar{X}}_c(\tau )$ denote the prevalence trajectories for host infectiousness without any interventions and with their combination, respectively. The combination trajectory now includes an IRS at $\tau =0$ and the MDA at variable time $\tau _0$, which may precede, be synchronous with, or follow the vector control campaign. Any value $\chi _s>1$ indicates a more effective, synergistic response to the interventions, and is the multiple for which the MDA deployed at $\tau _0$ is more (or potentially less) effective than the campaigns carried out in isolation. The ratio $\chi _s=2$ indicates twice as many infections were stymied by a given, scheduled programme; a synergy augmentation of two.

**Fig. 4:**
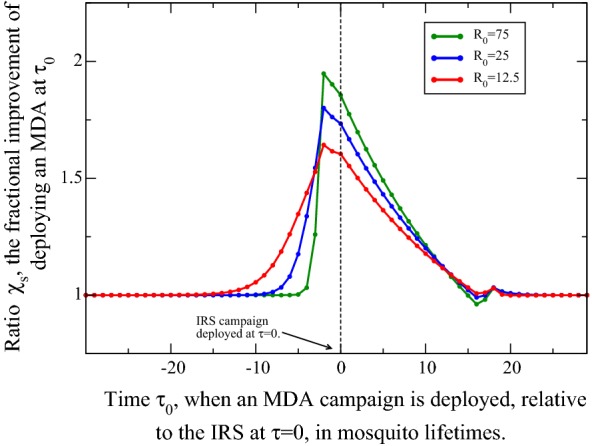
The fractional improvement $\chi _s$ of infection prevalence reduction, defined in Eq. 4, as a function of the time of MDA deployment, $\tau _0$. An impact of $\chi _s=2$ indicates the scheduling is twice as powerful in suppressing infections compared to isolated campaigns. The MDA timing is adjusted to that around a fixed IRS deployment scheduled at $\tau =0$, as indicated. A nearly synchronous deployment is most effective in suppressing infections/infectiousness in the populace, a trend even more apparent for high $R_0$. Nearly double the potency is possible in a high transmission setting with good campaign scheduling

For an MDA deployed at $\tau _{0}$ beginning well in advance of $\tau =0$, the time of the IRS campaign deployment ($\tau =0$), to well after, the impact ratio $\chi _s(\tau _0)$ is calculated for each set of trajectories and is plotted in Fig. 4. The ratio is shown as a function of this MDA deployment time, $\tau _{0}$, for a few indicated transmission settings. First, it is clear that both times far in advance and far after the IRS at $\tau =0$ asymptote to $\chi _s\rightarrow 1$, so that the impact of these timing sequences limits to that of the efficiency of isolated campaigns. The nearly synchronous campaigns modeled have a very strong complementary effect, especially at higher transmission. For $R_0=75$, the synergy is essentially double. This is, again, particularly important in considering programmatic design: with poor intervention scheduling, that is, by isolating these MDA and IRS campaigns, one is achieving somewhat less than half of their potential effect. Even in a stable, low transmission environment, $R_0=12.5$, one can achieve (better than) half-again the potency of the interventions simply by coordinating them. Given the programmatic choice of an MDA in year one with an IRS in year two, or both at the same time, the former appears to be a far less potent option. Such a choice negates all gains afforded by the synergy of these interventions.

Figure 4 also has a few interesting features in the profile. First, the maximum impact increases with transmission intensity. Second, the greatest impact is evident for an MDA campaign preceding the IRS schedule by exactly two mosquito lifetimes, $2g^{-1}$, that of the prescribed duration of the MDA chemoprophylaxis period (the time-period associated with the efficacy of the prescribed anti-malarial). Those gains established by the MDA campaign are propagated by the sustaining power of the IRS. In this efficient timing, an MDA campaign first causes an abrupt disruption in host infectiousness, with ${\bar{X}}$ instantly reduced to just 15% of pre-intervention value. Dynamics during the short course of the campaign attract the system briefly and weakly to the elimination point. Two mosquito lifetimes later, the MDA intervention expires, and host infectivity would begin resurgence and retract towards $X^*$ (${\bar{X}}\rightarrow 1$) except for the precisely instantaneous application of an IRS which sustains the established MDA gains. And they are yet improved by the marked reduction in vector population which mostly preempts subsequent transmission.

A quick comparison of this scheduling, that of an MDA applied a prophylactic period ($2g^{-1}$) ahead of an MDA, with the commensurate application of both MDA and IRS at $\tau =0$, indicates that the sustaining effect of this sequential ordering is only a slightly better suppressor of infections in the community than exactly synchronous deployment. Since these interventions are not carried out precisely in an instant in practice, synchronous campaign deployment would seem to be a good policy. This effect could however be significant in the case that a very long-lasting anti-malarial with strong chemoprophylactic effect is administered. In such a circumstance, the sequential deployment may prompt slightly higher gains, provided the IRS follows the MDA within the prophylactic period. In a practical sense, synchronous deployment is likely the best strategy.

**Fig. 5:**
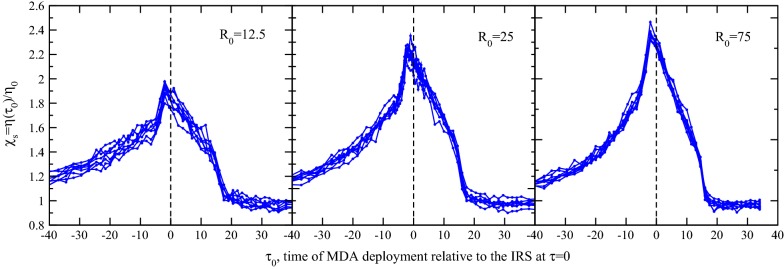
Simulation results for the fractional improvement $\chi _s$ in reducing host prevalence, defined in Eq. 4, as a function of the time of MDA deployment $\tau _0$. Each panel corresponds to a different, labeled reproductive number $R_0$ which is also taken to correspond with the annual EIR ${\mathcal {E}}_a$ [61]. The MDA timing is measured with respect to the IRS deployment at $\tau =0$, as indicated. It is again clear that a synchronous deployment, or just nearly so, preceding the IRS by the period of prophylaxis due to the MDA, is most effective in suppressing infections/infectiousness in the populace. The effect is double or better with good campaign scheduling

Before moving on, a curious feature near the conclusion of the IRS campaign on Fig. 4 is worth mentioning, where an MDA at the expiry of the effective period of the IRS performs slightly poorer than interventions applied in isolation (see $\tau _0\approx 16$ on the figure). At this point in time, the IRS is concluding and has made its full impact, and an MDA scheduled here therefore impacts the fewest infected individuals (see, for example, ${\bar{X}}$ at the conclusion of the IRS campaigns in Fig. 1). If instead the IRS expires fully prior to deploying the MDA, slightly more infectious individuals would be treated, and delays the onset of the rebound. This results in an incrementally greater overall impact, but only just better than the isolated campaigns. It is tepid; the period for really powerful gains using both interventions has essentially passed.

### Timing of IRS and MDA campaigns with *openmalaria*

The impact of these interventions and their timing in simulations is again assessed with the fractional improvement $\chi _s$, in Eq. 4 above, but for *openmalaria* simulations. Simulations are first run without interventions to establish ${\bar{X}}_0({\tau })$, the prevalence trajectory divided by its average value $X^*$, and separately with the MDA and IRS suitably isolated, determining ${\bar{X}}_{iso}(\tau )$. Additional simulations are run for a fixed IRS at $\tau =0$ and variable MDA at times $\tau _0$ before, during, and after the vector control campaign, establishing the relative impact $\chi _s(\tau _0)$ of Eq. 4. Results for eight trajectories are depicted in Fig. 5 for three different transmission intensities, demonstrating the absolute magnitude of the inherent stochastic noise.

**Fig. 6:**
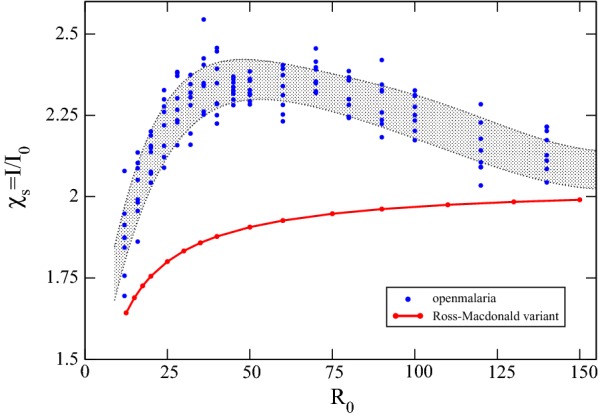
Impact $\chi _s$ for best-case scheduling, an MDA preceding an IRS campaign by the prophylactic period $2g^{-1}$, for different transmission intensities. In both simulation and the Ross–Macdonald variant, the impact of the interventions grows for small $R_0$, generally increasing to, or exceeding twice the impact of isolated campaigns. Interestingly, while the Ross–Macdonald model is monotonic, with growing impact for higher transmission intensities, *openmalaria* obtains a maximal impact and slowly wanes. Simulation data is plotted in blue (stochastic noise is apparent) and the grey highlighted background is drawn only to guide the eye; it has no numeric merit

As seen before in Fig. 4, an important attribute of Fig. 5 is the variable impact of the two interventions based on their relative timing. For MDA deployments well before or after the IRS at $\tau =0$, the impact decays towards that of isolated campaigns, $\chi _s\rightarrow 1$. The longer equilibration times in *openmalaria* merit a somewhat greater time window on the abscissa, though the general form and shape of the impact is impressively similar in both figures. In Fig. 5 as the campaigns overlap, their combined impact grows, suppressing more infections. The trend is markedly similar to that of the Ross–Macdonald impact plot, Fig. 4, peaking at an MDA deployment time just prior to the IRS campaign, and decays from this scheduling. The optimal deployment of one prophylactic period prior to the IRS is also reproduced: the same lesson is found in that the optimal strategy is to preserve the gains of the MDA by sustaining them with an immediately subsequent IRS. However, due to a latency in the vector response a shift in deployment time is required for an apt comparison with the initial killing impact of the IRS, see Appendix B. Furthermore, it is also clear from both Figs. 4 and 5 that MDA campaigns preceding the onset of the peak IRS killing effect are slightly more effective than the reverse. Just as in the Ross–Macdonald model, the maximum possible synergistic impact increases slightly with transmission intensity at these low to moderate intensities, and is nearly 2.5 times the baseline impact for $R_0=75$.

Next, an optimized MDA and IRS joint intervention is considered, and its maximal impact tracked for different transmission settings. Figures 4 and 5 both indicate their maximal impact grows slightly with the transmission intensity $R_0$. The campaign schedulings are fixed to that of an MDA preceding the IRS by a prophylactic period, $\tau _{MDA}=\tau _{IRS}-2$, and $R_0$ is varied through a larger range, running simulations and model trajectories for both. Plotted in Fig. 6 are profiles produced by both techniques, and show that the Ross–Macdonald variant has a more conservative prediction, as foreshadowed in the plots above, but also that *openmalaria* is interestingly rather non-monotonic. For transmission intensities of $\mathcal{E}_a\approx$ 25–50 bites/annum, the impact of the two campaigns is maximal, wanes thereafter, and likely asymptotes for higher transmission settings.

Except for the very lowest transmission intensities, synergistic deployment provides close to the double the impact of additive, separate campaigns for all $R_0$. Put another way, the choice to add a carefully timed MDA to an already scheduled mass vector control campaign has the potential to nearly triple the impact.

## Discussion

Given the powerful and complementary dynamic impacts that an MDA and IRS campaign exerts, respectively, on the human and vector population reservoirs of parasitaemia, it is logical to wonder whether significant synergies between carefully co-timed MDA and vector control interventions might exist. Such synergies have the potential to benefit control programmes struggling to make headway towards elimination in a constrained funding context.

In order to theoretically explore the nature and size of these potential synergies, a simple Ross–Macdonald variant with incorporated MDA and IRS interventions is explored, and a concise analysis of their overall impacts on population infection is carried out. Also included are *openmalaria* simulations in an effort to ensure that this effect and its size are not a feature of one modeling technique. The intent has been to uncover generalities that are scaling-level trends which are model-independent by nature.

The Ross/Macdonald theory has a number of limitations. Many complicating factors in both the modeled entomology and epidemiology are neglected in this formalism which in effect strips the elements of transmission down to a minimum. For example, not only are spatial considerations of transmission missing [62–64], but the mobility of both mosquito and host populations is absent [65–67]; imported parasites in the vector or hosts are not considered. Similarly, host immunity and super-infection are neglected, despite an oft-cited poor performance of the model in a high-transmission setting for this reason [68]. Also overlooked are potential heterogeneities in host selection, all but a few attributes of the vector(s), the role of parasite densities in transmission events, and in-host dynamics of infections. The mosquito population dynamics are restricted to maintain a constant emergence rate that replenishes the population lost with mortality rate *g*. Other than during the IRS campaign which kills mosquitoes by amplifying this rate $g\rightarrow \kappa g$, the mosquito population maintains an average value. More commentary on these complications (and others) can be found in reference [69]. Disregarding these influences, moderate (and stable [39]) transmission intensity environments might be most amenable and relevant to the simple transmission dynamics and model interventions here; infectious transactions in these settings may be assumed to be prevalent and several of these complications may play less of a role. Following the examination of the Ross/Macdonald variant are *openmalaria* simulations of MDA and IRS in order to demonstrate the strength and persistence of the observed effects in the presence of more complicating factors.

Vector control efforts are also considered to be effective. For instance, transmission is presumed to be largely dependent on endophagic vectors. Seasonal entomological trends are clearly also important for practical applications, but are neglected in order to focus on the effects of interventions in the absolute simplest of settings. Those geographies with highly-variable seasonality, with finite periods of malaria transmission in the calendar year prompted from annual rains absent other times, clearly require vector control to be deployed seasonally for a strong impact. Any accompanying MDA scheduling will likely also be similarly dominated by this seasonality, and may compete with their coordinated effect. While seasonal transmission may alter and compete with their coordination, this presents a situation that is beyond the scope of this investigation, which has instead focused on inter-intervention dynamics. It is possible that coordinated interventions, albeit perhaps not co-timed ones, may also predict large gains in seasonal transmission environments.

Simulations were carried out using intentionally simplified settings in order to unmask the fundamental transmission dynamics, and do not necessarily reflect a specific entomological and epidemiological environment. For example, vector biting in these simulations is carried out by a single species. There is also extremely weak case management and considerations of imported parasites in hosts or vectors are neglected. More sophisticated (and real) environments could be simulated but these embellishments might obscure the result. Case management, for example, is represented as a health system parameterization in *openmalaria* but functions as an additional intervention.

The Ross–Macdonald theory provides very useful insight into the mechanism of the IRS–MDA augmentation seen in both models. Each intervention is first modeled in isolation, with intensities of either of the interventions set to the same coverage and $R_0^I$ through the duration of the campaigns, an attempt to put them on equal ground considering transmission. Then, sweeping an MDA intervention’s timing beginning well prior to a vector control campaign to far after, a signature of intervention synergy is found when they are deployed very nearly together. A far greater impact, determined through the number of suppressed host infections, is found when an MDA is deployed nearly synchronously with an IRS campaign.

The modelled MDA campaign reduces the host parasite reservoir by 85%, and having a modest chemoprophylactic effect, protects the recipients for a short duration. After this, individuals are again susceptible and infections are re-established. The simple dynamics of the Ross/Macdonald model dictate that these infections restore an equilibrium, or balance of parasite transactions, post-intervention. The timeline to this re-equilibration, that of relaxing to the stable equilibrium point of freely exchanged parasites between the host and vector reservoirs, is simply related to the force of infection of the entomological setting the system resurges into. High transmission has a fast return to a proliferation of infections, a phenomenon explored in greater detail in our prior publication [20].

The IRS campaign impacts the vector, changing the effective entomological setting of the community. During the intervention, with a diminished mosquito population, the effective $R_0^I$ is reduced. The campaign’s longer duration of control, dictated by the effective period of the insecticide, inhibits the transmission of parasites by the vector during this period and leaves hosts with (potentially) no other means of purging infections except to clear them on their own slow timescale. Host infections/infectiousness wane very slowly but they have protection from new infections through the reduced vector population.

It is sensible that a strategy with combined campaigns would have more impact than isolated campaigns. If vector control can only offer the protection of reduced biting, while leaving individuals to fight infections off on their own, it is clear that a simultaneously deployed MDA offers an additional, and otherwise absent, therapeutic benefit. The timing analysis shows a characteristic signature of impact when both campaigns are deployed together, or nearly together, in time. Vector control and medical control campaigns affect different aspects of transmission, reducing the vector and host reservoirs, respectively, which gives them complementary, better-than-additive coordinated abilities to prevent new infections. Because the different reservoirs empty and refill at different dynamic rates, this complementarity is especially marked when vector and medical campaigns are deployed simultaneously, emptying both reservoirs at once and constraining the “refilling” to take place at the slower of the two dynamic rates.

This result is clear analytically from the Ross/Macdonald model but is also seen in the IRS and MDA *openmalaria* simulations of Fig. 2. Gains in eliminating host infections by the MDA are in both cases are prolonged by the vector control, just as is seen with the Ross/Macdonald formalism.

What is surprising is the size of the synergistic effect when these interventions are optimally deployed. Each intervention is powerful on its own, but in combination are extremely suppressive. The co-timed vector/medical campaign synergy is robust at all transmission intensities, but its exact trajectory varies by model, exhibiting an asymptotically-constrained increase with intensity for the simple model and a more complex non-monotonic response in *openmalaria*. In both models the synergy provides roughly twice the additive effect at high transmission intensities. The Ross/Macdonald model employs the most basic of transmission dynamics, contains essentially no elaborations, has very few parameters and indicates a strong synergy, together with full transparency of the forces and effects that enable it. The more complex *openmalaria* simulation, incorporating a much broader picture of transmission and many pertinent forces that shape it, confirms this synergy, and in fact predicts an even greater impact. These results, as they are general features of combined vector and medical control campaigns, are also likely to extend to vector and medical control campaigns with similar dynamic features.

## Conclusions

IRS campaigns are a widespread, globally ubiquitous method of vector control for malaria, used at all transmission intensities, and typically deployed via a mass campaign of limited duration. It is a potent transmission-suppressing intervention, if performed with high population coverage and effective insecticides. MDA is also a potent campaign-style transmission-suppressing intervention, but current WHO guidelines recommend its use primarily as an aid to elimination in very low transmission environments or for control in complex emergencies, while calling for operational research to further elucidate a potential role in programme planning [1, 36].

The co-use of MDA and IRS is explored, with both a semi-analytic model and *openmalaria* simulation. As elucidated by the simple model, the IRS/MDA campaign synergy is a consequence of the interplay of time scales between the two reservoirs of parasitaemia: the deep reservoir of infection in human hosts can be cleansed rapidly with dynamic MDA, but if the system tends towards a stable transmission equilibrium, will refill after the period of chemoprophylaxis afforded by the acting anti-malarial, and at a rate determined by the force of infection. Simultaneous deployment of vector control and MDA allows the human host reservoir to refill at the longer time scale determined by the insecticide used for vector control rather the shorter time scale of the anti-malarial, and this produces a profound and lasting suppression of prevalence even in high transmission environments, provided coverage of both interventions is high.

The synchronous or near synchronous deployment of these two interventions maximizes these better than additive gains; simultaneously deploying MDA together with an existing vector control campaign confers, in general, close to double the additive impact of the MDA and vector control campaign separated in time.

As MDA is a less common intervention than IRS, the synergistic benefit might also be expressed as the impact gained by adding an MDA to an existing vector control campaign as an accelerant. In *openmalaria*, the addition of an MDA is shown to amplify the impact of an IRS campaign by approximately four times its stand-alone impact. This is a striking result, given the enormous global investment in IRS.

This greater impact, if realized, could be translated into better health outcomes or cost savings. IRS is an intervention whose use is often limited by cost. An MDA campaign strategically co-deployed to maximize and amplify the effects of an IRS may reduce the number of spray rounds necessary to achieve a certain level of disease reduction, and therefore reduce an overall IRS investment. Widespread population LLIN deployment is typically performed campaign-style and shares many general features with the IRS modelled here. An MDA strategically applied to amplify an LLIN campaign may ensure the campaign achieves much higher impact than it would in the absence of MDA.

The potential benefit of MDA as a vector control accelerant should be researched and explored, though it should be noted that its function is entirely distinct from its use in an elimination setting. To deploy MDA in a high transmission setting over and over until elimination is achieved would be both costly and dangerous in terms of the development of drug resistance. MDA, used synchronously with IRS, or potentially LLINs, in a high transmission setting, may have a limited role to play in initially optimizing the impact of the vector control, and helping programmes reach steep reduction targets more easily and cost effectively. Cost savings in some scenarios may be very direct. In a high burden context, the treatment doses employed through MDA may prevent more infections than they consume.

Once prevalence is dramatically reduced, and provided that the new, achieved low in parasitaemia can be maintained without further campaign-style chemotherapy, MDA would presumably be halted until late pre-elimination, as per current guidelines.

Even in this limited sense as a vector control accelerant, MDA may not be palatable for control programmes or communities. Though it would be slightly more expensive, a highly sensitive, specific MSAT with newer diagnostics could be substituted for an MDA. Though it is not explicitly modelled here, a high coverage MSAT with a highly sensitive diagnostic will likely have a similar strong synergistic impact to an MDA if co-deployed with a vector control campaign. If such medical/vector co-campaign strategies are successful in greatly reducing prevalence within a short time, and provided that resurgence is prevented, they will also reduce community chemical exposure over time, and the exposures so concerning for resistance.

## Appendix A: Basic dynamics and the model interventions

### Dynamics, considerations of stability and scope

Transmission is determined by a simple, homogeneous Ross/Macdonald model variant which describes the dynamics of an infected/infectious subpopulation of hosts who infect ambient anopheles mosquitoes and vice-versa [46, 52]. The dynamics of the coupled infectious fractions are written,

5 $$\begin{aligned} \frac{d X}{d t} = mabZ\left( 1-X\right) -rX, \end{aligned}$$

6 $$\begin{aligned} \frac{d Z}{d t} = acX\left( e^{-gn}-Z\right) -gZ, \end{aligned}$$

for the proportions of infectious hosts *X* and mosquitoes *Z*. In the first expression, naive hosts of fraction $(1-X)$ become infected through coupled events at the rate *mabZ*, where $a^{-1}$ is an average time between human blood meals, *b* is the efficiency coefficient for mosquito-to-human transmission [70], and *m* is the population ratio of mosquitoes to humans. This first term is the creation rate of new infectious hosts from the naive host populace and is ignorant of the time lag of gametocyte development in hosts. Overlooking this latency, the infected and infectious proportions of humans are exactly equal. Host infections depart the population with the rate *r*, or have an average duration of $r^{-1}$. They are only cleared by waiting out this period (in the absence of medical treatment); deaths or migration are not considered. In the text, references to the “host reservoir” are synonymous to the proportion *X*, though they are not strictly equivalent.

The infectious proportion of mosquitoes *Z*, or sporozoite rate, is similarly constructed in Eq. 6, consisting of creation and annihilation rates. Mosquitoes die at the per-capita rate, *g*, which is the only means of diminishing the infectious vector population. New infectious mosquitoes are created at the rate *acX*, coupling to the infectious host proportion *X*, and *c* is a human-to-mosquito transmission efficiency. An extrinsic incubation period for infectiousness *n* is incorporated here by diminishing the eligible infected vector population so that only $P_e=\exp ({-gn})$ of newly infected anopheles survive to become infectious, a limitation based on the simple hazard model for mosquito survival. Incorporating the delay in this way, albeit crudely, again avoids distinguishing infected and infectious populations of mosquitoes, and the need to monitor them separately. Mosquitoes are assumed to carry infectiousness, once acquired, to their death. The fixed points of the dynamical system of Eqs. 5 and 6 are those of elimination, $X^*=Z^*=0$, and that of infection proliferation,

7 $$\begin{aligned} X^{*}=\frac{R_0-1}{R_0+\gamma }\quad \text {and,}\,\,\, Z^{*}=\left( \frac{\gamma P_e}{R_0}\right) \frac{R_0-1 }{1+\gamma },\,\,\,\text {with,}\,\,\, R_0=\frac{ma^2bce^{-gn}}{rg}. \end{aligned}$$

The definitions, $\gamma =ac/g$ and the reproductive number $R_0$ are introduced in these expressions. The former is introduced in the text and is the average number of self-inoculating bites a mosquito makes over its lifetime and $R_0$ represents the local intensity of transmission.

All trajectories for $R_0<1$ attract to the elimination point, ${\bar{X}}={\bar{Z}}=0$ and all others attract asymptotically to the stable, non-trivial equilibrium point $\{X^*,Z^*\}$ of Eq. 7. This is emphasized here because the control interventions considered here temporarily adjust the reproductive number, $R_0\rightarrow R_0^I$. With good coverage and effective chemoprevention and/or vector control, the reproductive number can fall below unity for the duration of the intervention (high effect size [48]). In this period, dynamics exist on a trajectory that attracts towards elimination but it will not result if retraction or intervention expiry causes only a temporary reduction in transmission. The rebounding, post-intervention $R_0$ re-introduces higher transmission and results in a dynamical resurgence to the equilibrium of Eq. 7, re-establishing widespread infections in the community.

Equations 5 and 6 describe a dynamical overview of the transmissive elements for infection and outline perhaps the simplest transmission model for host and vector dynamics. Infectious proportions are boosted by the coupled density transmission events and diminished with vector death or the expiry of host infections; these, again in the absence of interventions, are the only means which infections are gained or lost. The system above may be simplified with two transformations, first dividing the infectious populations by their (nontrivial) stable points, ${\bar{X}}=X/X^{*}$ and ${\bar{Z}}=Z/Z^{*}$, and second, scaling time by the mosquito lifetime $\tau =gt$. The system transforms to,

8 $$\begin{aligned} \frac{d{\bar{X}}}{d \tau }= \beta \left( \frac{Z^*}{X^*}\right) {\bar{Z}}(1-X^*{\bar{X}}) - \alpha {\bar{X}} \end{aligned}$$

9 $$\begin{aligned} \frac{d{\bar{Z}}}{d \tau }= \gamma \left( \frac{X^*}{Z^*}\right) {\bar{X}}(P_e-Z^*{\bar{Z}}) - {\bar{Z}}, \end{aligned}$$

and the following parameters have been introduced: $\alpha =r/g$ is the mosquito lifespan divided by the human infectious period, $\beta =mab/g$ is the lifetime average number of successful infecting bites per host, and $\gamma =ac/g$ is again the number of bites in a mosquito’s lifetime that infects it. The reproductive number is also simply expressed with the scaled parameters, $R_0=\gamma \beta P_e/\alpha =bcC/r$, which emphasizes its alternative interpretation which is that it is the ratio of two rates, that of infections invading the community $\gamma \beta P_e$, divided by the rate they leave, $\alpha$. The resulting dynamical system in Eqs. 8, 9 (and thus Eqs. 5 and 6) is in fact fully specified by just four independent parameters, $\{R_0,\gamma ,\beta ,P_e\}$. These are the transmission intensity of the setting $R_0$, the scaled interaction parameters for mosquito-to-human mediated transmission and vice-versa ($\gamma$ and $\beta$), and lastly $P_e=\exp ({-gn})$ is the proportion of mosquitoes that survive the incubation period. With the introduction of interventions below, their modelled effect will be to bottleneck transmission over a short period of time, reducing $R_0\rightarrow R_0^I$ through diminished mosquito-to-host or host-to-mosquito interactions.

An exceedingly desirable aspect of this modelling is that it is only a four parameter theory. The parameters are also remarkably coherent, consisting of the transmission intensity, the asymmetric transmission-mediating coefficients, and the extrinsic incubation period. As such a simple and transparent system, the mechanisms for intervention success, as well as those forces that restore (or potentially destabilize) equilibrium are simply illuminated and understood. In particular, this type of analysis affords a look at the fast/slow return to equilibrium post-intervention, and how it changes with transmission attributes.

### Interventions as finite periods of bottlenecked transmission

As outlined in the text, interventions are modelled as control efforts which temporarily change the reproductive number through (say) enhanced mosquito mortality or diminished human infectious periods. Effects such as these suppress mosquito-to-host transmission or vice-versa, and are modelled simply as short periods of bottlenecked transmission. For example, an MDA campaign has $r\rightarrow \xi r$ and $b\rightarrow b/\mu$, or $\alpha ^I=\xi \alpha$ and $\beta ^I=\beta /\mu$ (the superscript again denotes intervention-period values). All interventions are discussed in more detail in reference [20], which also describes how the augmented rates of $\xi$ and $\kappa$, as well as chemoprophylactic parameter $\mu$, are chosen while preserving the bottlenecked transmission environment specified by $R_0^I=0.5$. The periods of intervention activity/duration are approximated, and dynamical trajectories are calculated through the interventions, via Eqs. 8 and 9, but with dynamics determined by a reduced $R_0^I$ and associated parameters. When the intervention ends, the system dynamically relaxes according to the same ambient transmission conditions prior to the intervention, with a restored pre-intervention reproductive number $R_0$. Post-intervention the system regains the fixed points of Eqs. 7, where parasites are freely exchanged and measurable populations of infectious hosts and mosquitoes exist at modest $R_0>1$. This resurgence time mentioned in the text—the slow (or fast) re-acquisition of parasitaemia in the community—including the characteristic times of regaining equilibrium is explored in more detail in reference [20].

The time windows before, during and after an intervention are considered as highlighted in Fig. 1, and system dynamics are interpolated by matching conditions for the transitions between them. The first time period is a static trajectory, that of a community stuck at the equilibrium with stable endemic malaria. Evolving dynamics for this pre-intervention time period are elementary: parasites are exchanged freely from hosts to mosquitoes and vice-versa, and the parasite reservoirs are static. The second time window is that of the intervention which is brought about by an initiating impulse: the transmission environment is, in an instant, changed with the campaign. At the inception of the intervention, a proportion of the population (host or mosquito) affected has their parasite reservoir cleansed. This means, for example, when MDA is deployed and (say) 85% of a local population is treated, 85% of the infectious pool of humans has their parasite load cleared and their infectious status is exactly voided. This value of the infectious proportion is then used as an initial condition for the subsequent dynamics of the intervention period, with an associated set of reduced transmission parameters appropriate to the intervention. The time duration of this second period is estimated, which for an MDA is based on the duration of prophylaxis offered by the particular drug administered. Upon expiry of the intervention, system dynamics return to those of the time period prior to the intervention. The evolved infectious proportions at the expiry of the intervention period are used as initial conditions for the recovery stage, post-intervention. This is the third time window, the period of dynamical relaxation (which is a resurgence) subsequent to the intervention. Effects from a decaying intervention are not included, and, for example, insecticides do not wane in their potency: interventions are taken to be working or wholly ineffective. This level of analysis is performed for simplicity and robustness, choosing not to adorn or parameterize insecticides or individual pharmaceuticals. Their general effects of coverage, duration, and effective transmissive impact are ascribed for a given intervention.

All non-analytic and non-simulation results are full solutions to the coupled nonlinear Eqs. 8 and 9, integrated numerically with a fourth-order Runge–Kutta algorithm. Transmission parameter values are chosen in correspondence with previous work [47, 66, 71], which are $\gamma =0.642$, $\alpha =(150d)^{-1}/(10d)^{-1}$, and $P_e=1/e$.

## Appendix B: Details of ***openmalaria*** simulations

All simulations were run with the base model, which is often referred to as R0001 [53, 60]. The background and details of building and the determination of the integral 31 parameters which constitute the transmission model are detailed elsewhere [53–60, 72]. Transmission dynamics are discretized into 5-day timesteps for infectious/infectiousness status updates in hosts and mosquitoes, extrapolated parasite densities, forecasted acquired immunity, and severity of malarial episodes. Some of the dynamical ingredients present in the model for transmission in *openmalaria*, yet absent in the Ross/Macdonald variant, include superinfection, heterogeneous host selection and transmission, and health systems access and effects. Importantly, acquired partial immunity is also included and may be a result of a high number of prior episodes, and/or a history of high parasite densities, or maternal protection.

Separately, *openmalaria* enables the entomology and transmission elements dependent on the carrying capacity of the vector to be configured with an additional 16 parameters which specify the life and feeding cycle of the vector(s). Vector transmission dynamics are specified through a time-lagged deterministic difference equation [73, 74] and tethered to host transmission through the entomological inoculation rate (EIR). Transmission may be parameterized to be seasonal through the EIR but is here forced to be constant to afford a simple and direct comparison. A single vector species, *Anopheles gambiae*, whose parameters are chosen as previously specified [75] (in an included supplement) is considered for simulations here. The lone exception to these parameter assignments is that the extrinsic incubation period is set to $n = 10$ days in accordance with the average lifetime $g^{-1}$ mentioned in the text (which sets $e^{-gn}=e^{-1}$, and, for comparison it is $n = 11$ days in the reference).

A correspondence between the EIR, $\mathcal{E}$, and the reproductive number $R_0$ is needed to align the simulations with the semi-analytic model. Reference [61] details different effective $R_0$ values for *openmalaria* simulations with varying treatment probabilities. For the case of the base model without case management (essentially what is considered in the text), the reproductive number roughly corresponds with the annual EIR, or total bites per host per annum, $\mathcal{E}_a=(365d)\mathcal{E}$. To this end, simulations were run with annual EIR values in correspondence with the reproductive number of the Ross/Macdonald analytic model.

A very tepid health system is generically parameterized, exactly as detailed in the *openmalaria* wiki pages [76]. Defaults for simulations where case management is not a primary interest of the simulation are used, including those for access, adherence, and compliance. Uncomplicated cases of malaria are treated for only 4% of the populace and 48% of severe episodes are cured. Cure rates are unity for those who access care. This very low level of care allows reasonable correspondence with the cases of reference [61]. Other case fatality rates are as is typical as well as specified sequelae rates. These low rates of care also assist in a reasonable reestablishment of prior transmission environments post-intervention, something necessary for the analysis in the text.

Interventions are modelled as closely as possible with those described in the text for the semi-analytic model. The effects of the IRS do not slowly decay but are on or off through the use of a step function which activates the intervention from deployment for a duration of 36 timesteps, or $18\,g^{-1}$. The IRS is maximized in its mosquito killing impact, with preprandial and postprandial killing effects set to 0.99; deterrence is set to zero. The MDA is modelled as clearing the liver for 5 days and blood for 4 timesteps, or $2\,g^{-1}$. Coverage is 85% for both interventions, corresponding to the text. To compare the timing of interventions, the time index of initial intervention deployment is shifted for an apt comparison. This means, for an IRS deployed at ${\tau _0}$ in the Ross/Macdonald model, the sporozoite rate is instantaneously reduced at this moment (as detailed in the text). An equivalent *openmalaria* deployment is begun at the shifted time $\tau _0\,-\,$(15 days/10 days), accounting for an inherent latency in the sporozoite rate [73] to respond to the intervention. On this note, the MDA timing is also shifted. An MDA deployed within *openmalaria* at time $t_0=k\Delta t$ has its first effects at timestep $k+1$, and thus comparisons are made for a MDA deployed at $\tau _0$-(5d/10d) with a Ross–Macdonald campaign deployed at $\tau _0$. In brief, for reasons of simplicity, the timing of an IRS campaign is defined to be the very moment that the sporozoite rate plummets. *Openmalaria* takes about three timesteps (15 days) for this effect to transpire while it is, as designed, exactly immediate in the Ross–Macdonald intervention. This convention is mostly chosen for convenience: it might have instead matched the *openmalaria* standard, scheduling (for example) the Ross/Macdonald IRS with a full killing effect transpiring after a 15 days latent period.

Simulations are performed on a population of $N=10,000$ individuals with a demographic of that of Ifakara, as detailed in [54]. As none of the analytic results in the text above depend on demography, the distribution is rather irrelevant in this context, but necessary as the force of infection is dependent on this distribution in *openmalaria*. It was selected for generality and ease of reproducibility, and also reflects the general youthfulness of many sub-Saharan populations. The choice of population size is not selected with a community in mind but rather to simulate a large enough populace to have statistical relevance with small fractional random error. The multiple simulation results plotted demonstrate the modest stochastic variance in trajectories.

## Footnotes

1 The noted exception that an estimated 15–25 million children received seasonal malaria chemoprophylaxis [1, 77].
